# Validity of a visual analogue scale to measure and value the perceived level of sanitation: evidence from Ghana and Mozambique

**DOI:** 10.1093/heapol/czae092

**Published:** 2024-10-05

**Authors:** Ho Hei Cheung, Zaida Adriano, Bismark Dwumfour-Asare, Kwabena B Nyarko, Pippa Scott, Rassul Nala, Joe Brown, Oliver Cumming, Ian Ross

**Affiliations:** London School of Hygiene and Tropical Medicine, London WC1E 7HT, United Kingdom; WE Consult, 357 Avenida Patrice Lumumba, Maputo, Mozambique; Akenten Appiah-Menka University of Skills Training and Entrepreneurial Development, Sunyani Rd, Kumasi, Ghana; Kwame Nkrumah University of Science and Technology, Accra Rd, Kumasi, Ghana; WaterAid, 6th Floor, 20 Canada Square, London E14 5NN, United Kingdom; Instituto Nacional de Saúde, Av. Eduardo Mondlane 1008, Maputo, Mozambique; University of North Carolina at Chapel Hill, Chapel Hill 27599, United States; London School of Hygiene and Tropical Medicine, London WC1E 7HT, United Kingdom; London School of Hygiene and Tropical Medicine, London WC1E 7HT, United Kingdom

**Keywords:** Economic evaluation, visual analogue scale, sanitation, validity

## Abstract

Two billion people globally lack access to a basic toilet, and sanitation is a critical determinant of health and well-being. Evaluations of sanitation programmes typically measure disease or behaviour, and visual analogue scales (VASs) have not been used to measure users’ feelings about their level of sanitation. In this study, we assess the validity of a horizontal sanitation VAS numbered 0–10, with end anchors ‘best imaginable’ and ‘worst imaginable’ sanitation. In Kumasi, Ghana, we surveyed 291 participants before and after uptake of a container-based sanitation service. In Maputo, Mozambique, we surveyed 424 participants from treatment groups of a prior trial. We assessed construct validity by testing hypothesized associations between VAS scores and toilet characteristics and by respondents valuing three hypothetical sanitation states. We assessed responsiveness by comparing VAS with/without sanitation interventions. There was evidence (*P* < 0.05) for 60% of hypothesized associations in Ghana and 100% in Mozambique. For responsiveness, there was a 3.4-point increase (2.1 SD) in VAS 10 weeks post-intervention in Ghana and a 2.9 point difference (1.3 SD) in Mozambique. In valuation exercises, the mean was higher (*P* < 0.001) for the objectively better sanitation state. The sanitation VAS could be useful in economic evaluation to identify which improvements achieve quality-of-life gains most efficiently. For future studies, we recommend a vertical sanitation VAS numbered 0–100 with emojis at end anchors but retaining a 0–10 option for those who struggle with numeracy.

Key messages

- Participants scored their level of sanitation on a visual analogue scale (VAS) numbered 0–10, with end anchors ‘best imaginable’ and ‘worst imaginable’ sanitation.
- There was good evidence of construct validity (associations between VAS and toilet characteristics) and responsiveness (increases in VAS scores with interventions).
- Researchers and implementers could use the sanitation VAS as a rapid tool for evaluating the impact of interventions and in need assessments.

## Introduction

Evaluations of sanitation programmes often focus on disease outcomes (Wolf *et al*., 2022) and/or behaviour change (Garn *et al*., 2017). Health includes physical, mental and social well-being (WHO, 1948), but quantitative sanitation-focused research on these aspects of health beyond infectious disease has been limited (Sclar *et al*., 2018). This comes despite long-standing qualitative evidence that people particularly value outcomes such as privacy, safety and dignity in relation to sanitation (Elmendorf and Buckles, 1980; Cairncross, 1992; Mukherjee, 2001). Sustainable Development Goal target 6.2 focuses on ‘access to adequate and equitable sanitation and hygiene for all’ (UNICEF & WHO, 2023). Access is important, but its measurement tends to lead to a focus on attributes of toilets. For a full picture, it is important to measure people’s experiences too.

Impact evaluations are beginning to measure sanitation-specific dimensions of quality of life (Freeman *et al*., 2022; Ross *et al*., 2022). However, few studies have assessed participants’ overall evaluation of their sanitation status or satisfaction (Tumwebaze *et al*., 2013; Pickering *et al*., 2015), and none have assessed the validity of the measures used. An individual’s subjective perception of their level of sanitation overall not only could be complementary to more specific outcome measures but also provides information in its own right. Such measures might ask questions about ‘your level of sanitation’ or ‘satisfaction with your toilet’ on some scale. This requires the respondent to rapidly and unconsciously integrate multiple quality-of-life factors important to them, such as privacy, absence of smell, social status, safety and disease risk. A potential downside of focusing on satisfaction with a toilet is that it focuses attention on the infrastructure rather than the user. Innovative ways of assessing people’s experiences could inform not only academic studies but also sanitation policy and planning, whether as a rapid appraisal tool for existing perceived sanitation status in a population or as an indicative measure of progress.

A visual analogue scale (VAS) is a line with a rating scale on which respondents report an outcome, providing a measurable value function for ranking preferences (Dyer and Sarin, 1979). Intervals between placements on the line reflect differences in preference (Torrance, 1976). VAS scores represent strength of preference under certainty (Robinson *et al*., 2001), as opposed to the preferences under uncertainty typically used in health economic evaluation (known as Von Neumann–Morgenstern utilities) (Drummond *et al*., 2015).

VASs have been used in health economic evaluation since the 1970s (Patrick *et al*., 1973). They have varied with respect to the labels for the ends of the line (e.g. full health vs death), vertical or horizontal presentation and presence or absence of scale marks and numbers (Torrance *et al*., 2001). Health economists most commonly use VASs to measure a respondent’s subjective health status or to elicit a respondent’s valuation of hypothetical health states (Parkin and Devlin, 2006). The EuroQoL VAS (EQ-VAS), reproduced in Supplementary Material A, is a health status VAS used in over 40 000 studies to date (Devlin *et al*., 2020; Cheng *et al*., 2021), providing a measure of overall health-related quality of life. VASs have also been used to measure specific dimensions of health-related quality of life, such as pain (McCarthy *et al*., 2005).

The main advantages of VASs are their quick, inexpensive administration and amenability to self-completion (Torrance *et al*., 2001), whether in paper, digital or voice recognition surveys (Lundy and Coons, 2011). VASs have two main disadvantages when used in valuation (Torrance *et al*., 2001): first, context bias, whereby the VAS score for a state depends on the number of better and worse states presented at the same time (Bleichrodt and Johannesson, 1997), and second, end-aversion bias, whereby some respondents appear reluctant to select ratings near the end anchors (Streiner *et al*., 2015). However, adjustment for these properties is possible (Parkin and Devlin, 2006). A systematic review of the measurement properties of the EQ-VAS found it to have ‘sufficient’ construct validity in most populations but ‘sufficient’ test–retest reliability in fewer populations (Cheng *et al*., 2021).

In this study, we aim to assess the validity of a novel sanitation VAS for use in impact and economic evaluations, in the context of urban sanitation interventions in Mozambique and Ghana. In doing so, we address the need for validated and reliable sanitation measures focused on quality of life beyond disease, which can be simply and consistently deployed in studies and routine monitoring. When researchers measure outcomes in impact evaluations and economic evaluations, they need to be confident that they are truly measuring what they think they are measuring. Assessing validity is one means of providing that confidence or demonstrating that it is misplaced.

## Methods

We use data from two previous evaluations of urban sanitation interventions in Ghana (Tidwell *et al*., 2022) and Mozambique (Ross *et al*., 2022).

### Maputo setting and intervention

In Mozambique, 37% of the population had access to at least basic sanitation in 2022, up from 28% in 2000 (UNICEF & WHO, 2023). Maputo has a population of 1.1 million, with the majority living in basic settlements with unpaved roads. Our study site comprises low-income neighbourhoods in a 10 km^2^ area of the Nhlamankulu urban district, where the poorest people live in informally walled ‘compounds’ with many households in small single-storey dwellings sharing the same toilet. Low-quality pit latrines are common, often with squatting slabs made of wood or tyres and no water seal providing a barrier to smells and flies. Privacy can be a challenge since latrine walls are often made with scrap corrugated iron or plastic sheeting. The study design was observational. In 2019, 424 participants were recruited from intervention and control compounds of a prior non-randomized trial of a toilet subsidy programme (ClinicalTrials.gov, NCT02362932). Two people aged 18+ years were recruited per compound (one man and one woman) from different households. Intervention compounds were provided with a subsidized pour-flush toilet with a water seal. They discharged to a septic tank with soakaway, had a concrete superstructure and had metal doors lockable from the inside, all of which make them objectively higher-quality than control toilets. Compound inhabitants paid a 10–15% capital contribution. The study setting, intervention, sampling strategy and other methods are described in more detail elsewhere (Ross *et al*., 2022).

### Ghana setting and intervention

In Ghana, 29% of the population had access to at least basic sanitation in 2022, up from 20% in 2000 (UNICEF & WHO, 2023). Kumasi is Ghana’s second-largest city, with a population of 2.7 million. Independently operated pay-per-use public toilets, of which there are 400 around the city, are the primary sanitation facility for 36% of the city’s population (Ghana Statistical Service, 2013). Two-thirds of participants in a recent study in Kumasi had to walk at least 400 m to reach the nearest public toilet (Gaisie *et al*., 2018). Our study evaluated container-based sanitation (CBS) services provided by Clean Team Ghana (CTG), which rents out high-quality plastic toilets with a sealable internal waste container collected and replaced weekly. CBS is an alternative to traditional systems in densely packed informal settlements where people have low incomes and little space to construct household toilets (World Bank, 2019). It has the potential for higher user satisfaction than shared or public toilets, at affordable prices, but sector buy-in and financing are required to achieve scale in a sustainable way (Russel *et al*., 2019).

The design was a before–after enrolment study of CTG customers aged 18+ years, with participants surveyed shortly before installation of the toilet and 10 weeks afterwards. The study design focused on self-selecting customers, so no control group was used. Participants lived in 11 different Metropolitan and Municipal Assemblies (districts) within the Greater Kumasi Metropolitan Area. Housing was mixed, with some multi-storey tenement housing and some single-storey dwellings. Of the 404 people recruited at baseline in 2019, only 292 users completed both pre-CTG and post-CTG surveys. This attrition (28%) was due to customer dropout after installation (17%), customer dropout before installation (9%), and unavailability of active customers for the survey (2%). The study setting, intervention, sampling strategy and other methods are described in more detail elsewhere (Tidwell *et al*., 2022).

### Study design and VAS

**Figure 1.**
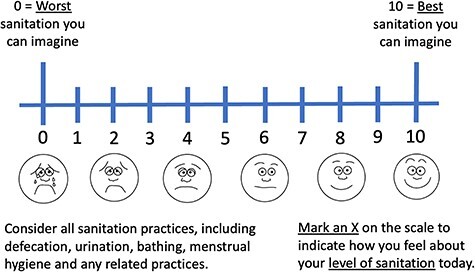
Sanitation VAS used in these studies

We use datasets from the aforementioned studies to explore the performance of a sanitation VAS (Figure 1), assessing its construct validity, responsiveness and convergent validity (explained further later). In Ghana, of 292 individuals interviewed at endline, 291 had VAS data at baseline (used for construct validity analyses) and 280 had VAS data for endline as well (used for responsiveness analyses). In Mozambique, all *n* = 424 individuals had VAS data. The sanitation VAS comprises a horizontal line, with 11 scale marks and numbers at every point from 0 to 10. Emojis were included to aid participants’ interpretation. This decision was informed by a pain VAS (Hawker *et al*., 2011) and by discussions during piloting in Mozambique, where fieldworkers agreed that it was more appropriate to have greyscale emojis than to reflect typical Mozambican skin tones or have yellow emojis. The end anchor for zero is ‘worst imaginable sanitation’, and that for 10 is ‘best imaginable sanitation’, adapted from the EQ-VAS (Cheng *et al*., 2021). In each setting, end anchors and guidance were depicted and explained in local languages (Portuguese in Maputo and Twi in Kumasi). We asked participants to rate on the scale how they felt about their ‘level of sanitation today’.

Note that, reflecting our results, we propose in Supplementary Material I an amended version of the VAS for use in future studies, rated 0–100 and with emojis only at end anchors. We also propose a simplified emoji version for people who struggle to understand the 0–100 scale (Supplementary Material I).

### Construct validity

The assessment of construct validity focuses on how well a measure reflects a concept (construct) that is not directly measurable (Fayers and Machin, 2015). To assess construct validity, we pre-specified hypotheses (Table 1) about the presence of associations between VAS scores and a set of toilet characteristics, drawing on the literature on motives for sanitation behaviours and well-being (Novotný *et al*., 2018; Sclar *et al*., 2018). We also included some negative controls (Arnold and Ercumen, 2016), hypothesized *not* to be statistically significantly associated with VAS score. In Ghana, we used the baseline dataset before the CBS toilet was delivered, since at endline, all were using CBS. We tested hypotheses in Stata 18 by regressing on VAS score including in turn each of the variables indicated in Table 1, per country. We also explored the consequences of accounting for covariance between the toilet characteristics, by regressing on all variables concurrently. We used linear regression with standard errors clustered at the district level in Ghana and compound level in Mozambique. In Ghana, we used wild cluster bootstrap inference because there were few clusters (Cameron *et al*., 2008). In Mozambique, all respondents lived in a small area of a single district, but the two people recruited per compound (from different households) usually shared the same toilet.

**Table 1.** Hypothesized associations of VAS scores with toilet quality variables

	Kumasi	Mozambique	Direction of hypothesized association (and rationale)
**Hypothesized to be associated with VAS score**			
Floor/slab	Toilet has ceramic pan/floor	Toilet floor is manufactured material^a^	Positive (more modern and easier to keep clean compared to lower-quality floors)
Water seal	Toilet has water seal	n/a (100% collinearity with intervention)	Positive (keep out smells and flies compared to direct-drop pit latrines)
Roof	n/a (96% have)^b^	Toilet roof is manufactured material^a^	Positive (stops people looking in from above and prevents rain entering)
Lock	n/a (97% have)^b^	Toilet locks from the inside	Positive (stops others entering, by mistake or on purpose)
Cleanliness	Toilet pan is not visibly dirty with faeces	Enumerator does not smell faeces	Positive (less disgusting to use)
Solid waste	n/a (not collected)	No solid waste observed around floor	Positive (less disgusting to use). nb, solid waste referred to waste other than anal cleansing materials
On-compound	Toilet is on-compound	n/a (100% on-compound)	Positive (easier and quicker to access, with more safety and less worry)
Handwashing	Handwashing facility near toilet	n/a (not collected)	Positive (easier to feel clean after using the toilet)
**Negative controls (hypothesized not to be associated with VAS score)**			
Years in dwelling	Respondent years lived in that dwelling		n/a (no obvious rationale for association with VAS score)
Education	Respondent completed primary education		
Partner	Respondent has a partner		

a Manufactured materials included cement, zinc sheets, tiles, etc.

b We only included variables if <85% of the sample was in a category to ensure a minimum of statistical power. Ideally, we would have tested for association with sharing the toilet, but almost all respondents in both samples shared their toilet with other households (Table 2).

### Convergent validity

Convergent validity explores whether two measures aiming to capture similar constructs are correlated (Fayers and Machin, 2015). We assessed convergent validity by correlation (Pearson’s *r*) between VAS scores and an index of sanitation-related quality of life (SanQoL-5). The SanQoL-5 index was developed in Mozambique (Ross *et al*., 2021b) and has now been used in several other countries. Its questions (Supplementary Material B) measure the respondent’s degree of achievement of five attributes: privacy, disgust, shame, disease and safety related to sanitation (Ross *et al*., 2021a). Higher SanQoL-5 index values represent better quality of life, with weighting of the five attributes arrived at via preference elicitation (Ross *et al*., 2021b). We hypothesized that VAS and SanQoL-5 would be positively correlated because they are capturing similar concepts. For context, correlation between the health-focused EQ-VAS and EuroQoL five-dimension index (EQ-5D) is typically around 0.5 (Whynes, 2008). For the Mozambique dataset, the sample design (two respondents per compound) permitted investigation of the convergence of VAS scores between users of the same toilet. This was achieved based on inter-rater reliability methods using the intracluster correlation coefficient (ICC) with a one-way random effects model (Koo and Li, 2016). The interpretation of this ICC is ‘fair’ (0.40–0.59), ‘good’ (0.60–0.74) or ‘excellent’ (>0.75) (Cicchetti, 1994). We expected that VAS scores would be strongly positively correlated (>0.5), but not completely correlated (<0.9), because two people may experience the same toilet differently.

### Responsiveness

Responsiveness is the ability of a measure to detect changes over time in the targeted construct (Fayers and Machin, 2015). We assessed responsiveness in Ghana by assessing the difference in VAS scores before/after the intervention. We assessed the similar concept of ‘known-groups validity’ in Mozambique, comparing intervention and control. Both analyses were done using Generalized Linear Mixed Models (GLMMs). We adjusted for sex, being aged over 60 years, and an asset-based wealth index, on the rationale that these covariates may be predictive of the participants’ responses to sanitation interventions (Ross *et al*., 2022). In Mozambique, the model is a two-level GLMM with standard errors clustered at the compound level. In Ghana, the model is a three-level GLMM with random effects at the individual and district level, and standard errors clustered at the district level. As a sensitivity analysis in Ghana, we also ran a linear regression with wild cluster bootstrap inference, which in Stata does not allow for three-level modelling. We report the effect size in SDs, a commonly used measure of responsiveness (Fayers and Machin, 2015).

### Validity in valuation

**Figure 2.**
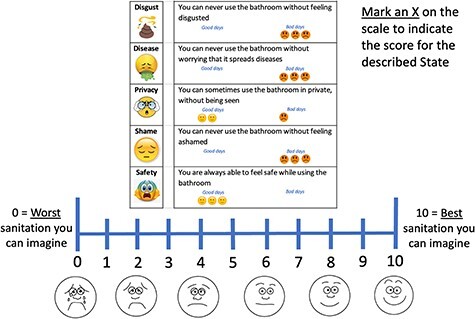
Example valuation card for a hypothetical sanitation state

In Ghana, a valuation exercise included in the study at baseline provided the opportunity to investigate another aspect of construct validity of the VAS. We developed hypothetical ‘sanitation states’ as combinations of SanQoL-5 attribute levels (example in Figure 2). At this point in the questionnaire, respondents had already answered SanQoL-5 questions and VAS for themselves, so were familiar with the concepts. On the cards, each attribute was visualized with an emoji, and each attribute level (e.g. always, sometimes, rarely and never) was visualized by a number of happy/unhappy emojis. Fieldworkers first explained the SanQoL-5 card and then asked the respondent to value the state on the VAS. Two further states were also valued (Supplementary Material C). We hypothesized that the SanQoL-5 state, which is objectively better than the other two, would have a higher mean VAS score in paired *t*-tests.

### Ethics

The Mozambique study received prior approval from the Comité Nacional de Bioética para a Saúde (ref: IRB00002657) at the Ministry of Health in Mozambique. The Ghana study received prior approval from the Committee on Human Research, Publications and Ethics at the Kwame Nkrumah University of Science and Technology (ref: CHRPE/AP/317/19). The protocol for the present study was reviewed by the MSc Research Ethics Committee at the London School of Hygiene and Tropical Medicine, which concluded that additional ethics approval was not required (ref: 27732).

## Results

### Sample characteristics

In Ghana, most respondents were female (77%), while in Mozambique about half were female (52%) (Table 2). Ghanaian respondents were slightly older on average (44.1 years) than Mozambican respondents (39.9 years). The average household size was higher in Mozambique (5.1) than in Ghana (3.2). Almost all Mozambican participants had on-premises piped water (98%), while only 23% of Ghanaian participants did. In Mozambique, participants were equally split between users of pit latrines and users of pour-flush toilets, and almost all were using a toilet which was shared with other households (89%) and/or which was on plot (98%). In Ghana, most participants relied on pay-per-use public toilets (72%) at baseline, and 83% used flush or pour-flush toilets. We do not report statistical tests comparing the two samples in Table 2 because differences between them are not relevant to our analyses, and validity is best assessed across diverse populations. Differences between groups compared in Mozambique are reported and discussed elsewhere (Ross *et al*., 2022). In Ghana, dropouts were not significantly different from non-dropouts in terms of sex, VAS and SanQoL-5, using a *t*-test (Supplementary Material D).

**Table 2.** Sample characteristics for datasets used in construct validity analyses

	Ghana at baseline (*n* = 291)	Mozambique (*n* = 424)
**Respondent demographic characteristics**		
Respondent is female	215 (77%)	220 (52%)
Respondent mean age, years	44.1 (12.7)	39.9 (15.3)
18–29	14 (5%)	126 (30%)
30–44	147 (51%)	155 (37%)
45–59	84 (29%)	88 (21%)
60+	46 (16%)	55 (13%)
Household size	3.2 (1.7)	5.1 (3.0)
Completed primary school or above	192 (67%)	268 (63%)
Piped water on premises	63 (23%)	416 (98%)
**Sanitation characteristics**		
Type of toilet		
Flush or pour-flush toilet	223 (83%)	222 (52%)
Pit latrine	47 (17%)	202 (48%)
Nature of sharing		
Not shared with other households	13 (5%)	47 (11%)
Shared but not public toilet	62 (23%)	377 (89%)
Public toilet	195 (72%)	0 (0%)
Toilet is on plot	42 (16%)	416 (98%)
Toilet has solid walls	205 (74%)	275 (65%)
Toilet has inside lock	182 (66%)	187 (44%)

Data are *n* (%) for categorical variables and mean (SD) for numerical variables. Percentages for categorical variables are % of those with data for that variable.

### Validity

When hypotheses for the Ghana sample were assessed individually (Table 3), there was evidence at the 10% level for 80% of posited associations between VAS scores and toilet characteristics (60% at the 5% level). For the Mozambique sample, there was evidence at the 10% level for 100% of hypotheses (100% at the 5% level). When assessed concurrently, there was evidence at the 10% level for 60% of hypotheses in Ghana (40% at the 5% level) and 100% in Mozambique (80% at 5% level). In neither country were any of the negative controls associated with VAS scores at the 10% level.

**Table 3.** *P*-values on coefficients for hypothesized associations in regression, individually and concurrently

	Ghana		Mozambique	
	Individual	Concurrent	Individual	Concurrent
**Hypothesized to be associated with VAS score**				
Floor/slab	0.054*	0.852	<0.001***	0.007***
Water seal	0.006***	0.040**	n/a	n/a
Roof	n/a	n/a	<0.001***	0.069*
Lock	n/a	n/a	<0.001***	0.007***
Cleanliness	0.004***	0.112	<0.001***	0.010**
Solid waste	n/a	n/a	<0.001***	0.009***
On-compound	0.214	0.066*	n/a	n/a
Handwashing	<0.001***	<0.001***	n/a	n/a
R^2^		22%		34%
**Negative controls**				
Years in dwelling	0.266	n/a	0.484	n/a
Education	0.312	n/a	0.295	n/a
Partner	0.232	n/a	0.854	n/a

All associations were in the hypothesized direction. Individual models regressed on VAS scores and the indicated variable. Concurrent models regressed on all hypothesized variables at once.

* , ** and *** indicate significance at the 10, 5 and 1% level. Pairwise correlations between variables are tabulated in Supplementary Material C. Detailed regression output, including coefficient values, is in Supplementary Material F.

Correlation between VAS scores and SanQoL-5 index was 0.51 (*P* < 0.001 against H_0_ of no correlation) in Mozambique and 0.70 in Ghana (*P* < 0.001), similar to correlations identified between the EQ-VAS and EQ-5D (Whynes, 2008). Distributions of VAS scores and SanQoL-5 index values with/without intervention are provided in Supplementary Material G. In Mozambique, the ICC was 0.78 for convergence of VAS between two respondents (from different households) using the same toilet on the same compound, indicating substantial but not complete correlation as hypothesized.

### Responsiveness

**Figure 3.**
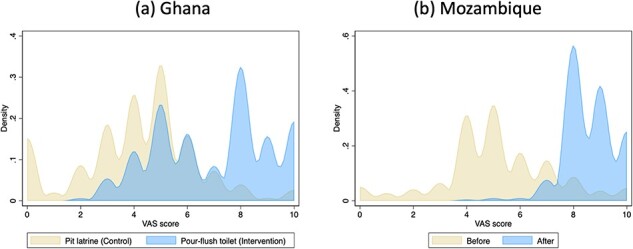
Probability density distributions of VAS scores by treatment group

There was good evidence for responsiveness (*P* < 0.001), with a 3.4-point increase in VAS scores (95% CI: 3.2–3.6) in Ghana after the intervention. The wild cluster bootstrap model gave the same point estimate at *P* < 0.001. In Mozambique, there was a 2.9-point difference (95% CI: 2.4–3.4) in VAS scores between the intervention and control groups (*P* < 0.001). Full regression output is in Supplementary Material H. Distributions of VAS scores by treatment group clarify the pattern of responses driving these results (Figure 3). Effect sizes were large at 2.1 SD in Ghana and 1.3 SD in Mozambique. Limitations of study designs for causal inference are explored in the Discussion section—the important result here is that the VAS was responsive, irrespective of bias.

Distributions are presented as probability density, but note that the VAS used in these studies was an 11-point rating scale ranging from 0 to 10. Histograms of VAS score in the overall sample per country are in Supplementary Material G.

### Valuation

Participants valued three states (Table 5), of which State 3 is objectively preferable to the other two. State 3 is preferable to State 1 for four out of five attributes. State 2 is only preferable to State 3 for one attribute, and State 3 is preferable on the other four. In line with our hypotheses, valuations for State 3 were significantly higher (*P* < 0.001) in paired *t*-tests than for States 1 and 2. For State 1 and State 2, which are relatively similar in terms of overall attribute levels, the 95% CIs of means overlap.

**Table 5.** Mean VAS valuations of three SanQoL states (*n* = 291)

	State 1	State 2	State 3
Disgust	Rarely	Never	Sometimes
Disease	Never	Never	Sometimes
Privacy	Rarely	Sometimes	Always
Shame	Never	Never	Sometimes
Safety	Rarely	Always	Rarely
Mean	4.2	4.4	5.5
(95% CI)	(4.1–4.3)	(4.2–4.5)	(5.1–5.8)

A sanitation state is a combination of SanQoL-5 attribute levels, where ‘never’ for all five attributes is the worst-possible state and ‘always’ for all five is the best-possible (Supplementary Material B).

## Discussion

In this study, we have evaluated different aspects of validity of a sanitation VAS on which respondents indicate how they feel about their ‘level of sanitation today’ on a 0–10 scale with emojis. There was evidence at the 5% level for 60–100% of hypothesized associations between VAS scores and toilet characteristics when assessed individually and 40–80% when assessed concurrently. There was good evidence for responsiveness, with effect sizes on VAS scores of 1.3–2.1 SD associated with sanitation interventions, which included the objective level of service substantially. We observed convergence between VAS scores and SanQoL-5 index values and between VAS scores of two respondents using the same toilet. To our knowledge, this study represents the first exploration of the validity of a sanitation VAS and one of the few studies of the validity of sanitation measures focused on quality of life.

It was surprising that the variable for ‘toilet is on-compound’ (as opposed to having to leave the compound) was not significant as an individual association in Ghana (Table 3). However, that variable negatively correlated with ‘ceramic pan/floor’ and ‘water seal’ variables (Supplementary Material E), suggesting that on-compound toilets were more likely to be of low-quality, which potentially drives this result. It also explains why someone who already has an on-compound toilet might want to invest in CBS as an upgrade. Accounting for these and other variables in the concurrent regression, the on-compound variable becomes significant at the 10% level with a positive coefficient, as hypothesized (Supplementary Material F). In construct validity analyses, what is important is not so much the relative size of coefficients (reported in Supplementary Material F), but rather the fact that associations are present in the hypothesized direction and significant (Reeve *et al*., 2013).

The benefits of sanitation beyond infectious disease are likely to underpin household willingness to pay for sanitation improvements. These benefits are diverse (Novotný *et al*., 2018), and different outcomes are important to different people. Global measures such as VAS can capture an overall picture of how people feel about their situation with respect to an outcome (Parkin and Devlin, 2006). Measuring toilet characteristics objectively has often been the focus of efforts to assess sanitation quality (Tidwell *et al*., 2018; Schelbert *et al*., 2020). Measures like the sanitation VAS focus on the subjective experience of the individual. This is important because sanitation interventions might improve infrastructure and/or behaviours, but with no impact on quality-of-life outcomes.

The sanitation VAS has practical applications in impact evaluation. Our responsiveness analyses illustrate how different sanitation programmes might be compared with respect to their effectiveness for the self-perceived level of sanitation. VAS may not be an obvious candidate for a primary outcome in an impact evaluation, but it could be a useful addition to the armoury of secondary outcomes to give a more rounded picture of comparative effectiveness. In the broader Mozambique study, e.g. the evaluated intervention was found to have no effect on health outcomes such as diarrhoea or stunting after 24 months (Knee *et al*., 2021). However, it did have a substantial impact on VAS (Table 4), providing complementary information about different dimensions of effectiveness important to both decision-makers and toilet users. The simplicity of the VAS and its amenability to self-completion could make it particularly useful in surveys based on phone messages. Just as doctors use VAS for rapid assessment at the bedside (Bailey *et al*., 2012; Lvovschi *et al*., 2021), it is possible to imagine VAS being used as a rapid diagnostic at the community level.

**Table 4.** Evidence for responsiveness between groups with/without an intervention in GLMM regressions

		Ghana (*n* = 280)		Mozambique (*n* = 424)	
		Without intervention	With intervention	Without intervention	With intervention
Sample size		280 (before)	280 (after)	202 (control)	222 (int’n)
Mean VAS score	Mean	5.1	8.6	4.1	7.0
	(SE)	(0.1)	(0.1)	(0.2)	(0.1)
Unadjusted models	Unadjusted difference. (95% CI)	3.4*** (3.2–3.6)		2.9*** (2.4–3.4)	
	*P*-value	<0.001		<0.001	
Adjusted models	Adjusted difference (95% CI)	3.4*** (3.2–3.6)		2.9*** (2.4–3.4)	
	*P*-value	<0.001		<0.001	
	Effect size (Cohen’s *d*)	2.1 SD		1.3 SD	

In Ghana, the model is a three-level GLMM with random effects at the individual and district level, with standard errors clustered at the district level. In Mozambique, the model is a two-level GLMM with standard errors clustered at the compound level. Adjusted models include gender, aged 60+ years and wealth index as covariates.

* , ** and *** indicate significance at the 10, 5 and 1% level. Detailed regression output is in Supplementary Material F.

The sanitation VAS also has applications in economic evaluation and in understanding preferences (Ross, 2022), since it provides a measurable value function whereby intervals between placements reflect differences in preference (Torrance, 1976). These properties are useful in understanding the relative value of states of the world. We present results of such an application in valuing SanQoL-5 states (Table 5). However, VAS could equally be used to understand the relative value of specific sanitation service options (e.g. pit latrine versus pour-flush toilet) or other service characteristics (e.g. public toilet at 5-min round trip vs 10 or 15 min). Other analyses might explore the relative contribution of improvements in toilet characteristics to increases in VAS scores to identify where the largest incremental gains might be made at low cost.

In using VAS to understand preferences, it should be noted that VAS valuations represent decisions under certainty rather than uncertainty (Drummond *et al*., 2015). Therefore, while they can correctly measure strength of preference, VAS valuations cannot be called ‘utilities’ without strong assumptions about respondents being risk-neutral (Dyer and Sarin, 1979). However, the health VAS literature tends to support the idea that this is not such a problem (Parkin and Devlin, 2006) depending on the purpose and may be an advantage for social decision-making purposes. While VAS scores can be a substitute for multiattribute utility indices for some purposes (Pickard *et al*., 2007), this is unlikely to be the case for all purposes (Rashidi *et al*., 2006). As far as sanitation is concerned, then it makes sense to use the SanQoL-5 index and sanitation VAS concurrently.

Comparing SanQoL-5 scores and VAS scores among the same individuals, a slight end-state aversion is seen (Parkin and Devlin, 2006). For example, respondents tend to avoid selecting a score of 10 (Figure 3), even when they have the highest possible levels for all SanQoL-5 questions. In the ‘with-intervention’ distributions for both Ghana and Mozambique, the modal VAS score is 8, whereas the modal SanQoL-5 index value is 1.0 (Supplementary Material G), which reflects the pattern observed between EQ-VAS and EQ-5D (Whynes, 2008). The two outcomes measure similar but slightly different things, so are complementary.

Given the evidence in support of validity we have presented and the relative simplicity and directness of the question, the sanitation VAS is likely to be appropriate for general use. However, the assessment of validity is an ongoing process and, in particular, an exploration of people’s cognitive processes when interpreting and responding to the sanitation VAS would be helpful, e.g. a think-aloud study (Ernstsson *et al*., 2020). Such an assessment could also explore alternative formulations of the question and end anchors. Having subsequently piloted and applied VAS in other counties and populations (manuscripts under preparation), we recommend future studies use a vertical 0–100 scale to allow more granularity, with emojis applied only at end anchors to avoid the risk of the emojis being used as a heuristic to ignore the numeric scale. The proposed new formulation, depicted in Supplementary Material I, is closer to a truly continuous analogue scale (Price *et al*., 2012). However, a fully emoji-based alternative may be necessary for respondents (a minority in most populations) who struggle to understand numeric scaling, which can occur with both 0–10 and 0–100 versions (also provided in Supplementary Material I).

Our study does have some limitations. First, the evidence we have provided is from two quite specific contexts alongside evaluations of interventions. It is therefore hard to draw conclusions about why the results were slightly different in Ghana and Mozambique, except to the extent that Mozambique was a trial-based sample, so had a large variation in toilet types between the two arms. In future research, it would be important to understand the properties of the sanitation VAS in the general population, especially rural areas. The specificity of these datasets is also a limitation to the construct validity assessments, which would ideally be repeated in a sample of people using a variety of different types of sanitation and in representative samples of larger populations. This would also allow more hypotheses to be tested, e.g. the role of sharing. Second, the effect sizes in the responsiveness analyses should be compared with caution, since the two samples we draw on differ in important characteristics (Table 2), and the study designs and interventions are also different. Notably, the Ghanaian sample comprised self-selecting individuals who had signed up to the CTG service, whereas the Mozambican sample was drawn from trial-enrolled compounds. The Ghana study has no control group and so is likely to have greater risk of bias than the Mozambique study, and further limitations regarding causal inference are discussed in the respective parent study papers (Ross *et al*., 2022; Tidwell *et al*., 2022). However, the focus of the present study is to assess responsiveness of the VAS rather than comparing effect sizes between interventions, and our study design is appropriate for this purpose. Third, only certain aspects of validity and reliability could be tested given the data available in the underlying studies. Future studies would ideally investigate test–retest reliability, namely, whether someone’s VAS score is sufficiently stable in the short term, e.g. comparing two measurements 7–14 days apart (Polit, 2014), which was not possible in these studies.

There are also limitations to the VAS itself. First, the fact that people incorporate different subjective weightings of sanitation-related outcomes into their VAS response is a limitation to the extent that two people’s responses may be based on different things. One person may base their response primarily on their recent experience of disgust, while another might base it primarily on privacy. However, this is also a strength in that it allows people to weigh up what is important to them, in the same way as questions about life satisfaction and happiness do (Welsch, 2006). Second, it is inherently challenging to compare subjective self-reported outcomes between individuals, such as pain or happiness. Any VAS, such as the sanitation VAS or EQ-VAS, has the same challenges. However, they should at least behave similarly in different populations, and we have only evaluated VAS in two settings here. Comparing the validity of VAS in further more diverse settings, ideally in representative samples of larger populations, would allow claims of cross-cultural validity to be made. Third, and related, there is risk of reporting bias with any self-reported outcome, which is difficult to measure and account for. In both countries, field teams stated that they were not linked to the implementing non-government organisation, but participants may have wanted to appear grateful. In neither country were participants getting the service for free—in Ghana, participants were normal customers paying the standard price, and in Mozambique, there was a 10–15% capital contribution. However, the risk remains that the ‘with-intervention’ estimates are upwardly biased and the responsiveness of VAS is overestimated.

Third, an individual’s evaluation of their current sanitation status is affected by ‘adaptation’ to previous experience of toilets (Nussbaum, 2001). Most people globally, though not all, are likely to have experienced ‘full health’ for some part of their life. However, fewer people will have experienced the best level of sanitation they can imagine (Figure 1), and people vary with respect to their imagination and their prior experience. An evicted tenant who has moved from using a high-quality toilet to a low-quality one (in their new home) may attribute a lower VAS score to their current status than someone who has only ever used a low-quality toilet, due to the relative deprivation compared to their prior experience.

When evaluating a policy’s effectiveness on an outcome, we need to be confident that our measurement of that outcome is truly capturing what we think it is. This is especially true if we are asking policymakers to make decisions based on findings. For example, estimated prevalence of diarrhoea (Rego *et al*., 2022) and handwashing behaviour (Ram *et al*., 2014) are sensitive to the methods used to measure them. More researchers, in the sanitation field and in general, could explore the validity and reliability of outcome measures they use.

## Conclusion

A sanitation VAS provides a simple and rapid means of capturing a respondent’s self-perceived level of sanitation, and we have provided evidence in support of its validity in two settings. VASs are widely used in health impact evaluation and economic evaluation, and they have potential for similar applications in the field of sanitation. They can inform not only academic studies but also sanitation policy and planning, whether as a rapid appraisal tool for existing perceived sanitation status in a population or as an indicative measure of progress. Benefits of sanitation beyond infectious disease are likely to underpin willingness to pay for toilets and later upgrades, so it is important to understand broader outcomes as part of efforts accelerate towards universal access. Global measures such as VAS can capture an overall picture of how people feel about an outcome, focusing on the subjective experience of the individual. Future research priorities for VAS are assessing test–retest reliability and exploring construct validity in contexts with more diverse toilet types and larger samples.

## Supplementary Material

czae092_Supp

## Data Availability

Datasets and Stata code are available open access online at https://doi.org/10.17605/OSF.IO/CX5AV.

## Reflexivity statement

This work was based on H.H.C.’s MSc thesis focused on the Ghana data, so she has the first author position. I.R. supervised H.H.C., the Mozambique data were collected as part of his PhD, and he designed the VAS aspects of the Ghana study, so he has the last author position. I.R. is the corresponding author because H.H.C. was an MSc student and has not stayed in academia. All other authors contributed to either the Mozambique or Ghana studies from which data were drawn. Z.A. and R.N. are from and based in Mozambique. B.D.A. and K.N. are from and based in Ghana. Of the nine authors, four are women.

## References

[R1] Arnold BF, Ercumen A. 2016. Negative control outcomes: a tool to detect bias in randomized trials. *JAMA* 316: 2597–8. 28027378 10.1001/jama.2016.17700 PMC5428075

[R2] Bailey B, Gravel J, Daoust R. 2012. Reliability of the visual analog scale in children with acute pain in the emergency department. *Pain* 153: 839–42. 22305630 10.1016/j.pain.2012.01.006

[R3] Bleichrodt H, Johannesson M. 1997. An experimental test of a theoretical foundation for rating-scale valuations. *Medical Decision Making* 17: 208–16. 9107617 10.1177/0272989X9701700212

[R4] Cairncross S . 1992. Sanitation and water supply: practical lessons from the decade. *Water and Sanitation Discussion Paper Series* 71: 101–2.

[R5] Cameron AC, Gelbach JB, Miller DL. 2008. Bootstrap-based improvements for inference with clustered errors. *The Review of Economics and Statistics* 90: 414–27.

[R6] Cheng LJ, Tan RL, Luo N. 2021. Measurement properties of the EQ VAS around the globe: a systematic review and meta-regression analysis. *Value in Health* 24: 1223–33. 34372988 10.1016/j.jval.2021.02.003

[R7] Cicchetti DV . 1994. *Guidelines, Criteria, and Rules of Thumb for Evaluating Normed and Standardized Assessment Instruments in Psychology*, Vol. 6. USA: American Psychological Association, 284–90.

[R8] Devlin N, Parkin D, Janssen B. 2020. *Methods for Analysing and Reporting EQ-5D Data*. Cham, Switzerland: Springer. 33347096

[R9] Drummond M, Stoddard GL, Torrance GW. 2015. *Methods for the Economic Evaluation of Health Care Programmes*, 4th edn. Oxford, UK: Oxford University Press.

[R10] Dyer JS, Sarin RK. 1979. Measurable multiattribute value functions. *Operations Research* 27: 810–22.

[R11] Elmendorf M, Buckles PK. 1980. Sociocultural aspects of water supply and excreta disposal (volume 5). World Bank.

[R12] Ernstsson O, Burstrom K, Heintz E, Molsted Alvesson H. 2020. Reporting and valuing one’s own health: a think aloud study using EQ-5D-5L, EQ VAS and a time trade-off question among patients with a chronic condition. *Health and Quality of Life Outcomes* 18: 388. 10.1186/s12955-020-01641-4 PMC7745504 33334348

[R13] Fayers P, Machin D. 2015. *Quality of Life: The Assessment, Analysis and Interpretation of Patient‐Reported Outcomes*, 3rd edn. Hoboken, NJ, USA: Wiley.

[R14] Freeman MC, Delea MG, Snyder JS et al. 2022. The impact of a demand-side sanitation and hygiene promotion intervention on sustained behavior change and health in Amhara, Ethiopia: a cluster-randomized trial. *PLoS Global Public Health* 2: e0000056. 10.1371/journal.pgph.0000056 PMC10021625 36962125

[R15] Gaisie E, Poku-Boansi M, Adarkwa KK. 2018. An analysis of the costs and quality of infrastructure facilities in informal settlements in Kumasi, Ghana. *International Planning Studies* 23: 391–407.

[R16] Garn JV, Sclar GD, Freeman MC et al. 2017. The impact of sanitation interventions on latrine coverage and latrine use: a systematic review and meta-analysis. *International Journal of Hygiene and Environmental Health* 220: 329–40. 27825597 10.1016/j.ijheh.2016.10.001 PMC5414716

[R17] Ghana Statistical Service . 2013. 2010 population & housing census: national analytical report.

[R18] Hawker GA, Mian S, Kendzerska T, French M. 2011. Measures of adult pain: Visual Analog Scale for Pain (VAS Pain), Numeric Rating Scale for Pain (NRS Pain), McGill Pain Questionnaire (MPQ), Short-Form McGill Pain Questionnaire (SF-MPQ), Chronic Pain Grade Scale (CPGS), Short Form-36 Bodily Pain Scale (SF). *Arthritis Care & Research* 63: S240–52. 22588748 10.1002/acr.20543

[R19] Knee J, Sumner T, Adriano Z et al. 2021. Effects of an urban sanitation intervention on childhood enteric infection and diarrhoea in Mozambique. *eLife* 10: e62278. 10.7554/eLife.62278 PMC8121544 33835026

[R20] Koo TK, Li MY. 2016. A guideline of selecting and reporting intraclass correlation coefficients for reliability research. *Journal of Chiropractic Medicine* 15: 155–163. 27330520 10.1016/j.jcm.2016.02.012 PMC4913118

[R21] Lundy JJ, Coons SJ. 2011. Measurement equivalence of interactive voice response and paper versions of the EQ-5D in a cancer patient sample. *Value in Health* 14: 867–71. 21914508 10.1016/j.jval.2011.03.001

[R22] Lvovschi VE, Hermann K, Lapostolle F, Joly LM, Tavolacci MP. 2021. Bedside evaluation of early VAS/NRS based protocols for intravenous morphine in the emergency department: reasons for poor follow-up and targeted practices. *Journal of Clinical Medical* 10: 5089. 10.3390/jcm10215089 PMC8584399 34768612

[R23] McCarthy M Jr, Chang CH, Pickard AS et al. 2005. Visual analog scales for assessing surgical pain. *Journal of the American College of Surgeons* 201: 245–52. 16038823 10.1016/j.jamcollsurg.2005.03.034

[R24] Mukherjee N . 2001. Achieving sustained sanitation for the poor: policy and strategy lessons from participatory assessments in Cambodia, Indonesia and Vietnam. World Bank.

[R25] Novotný J, Hasman J, Lepič M. 2018. Contextual factors and motivations affecting rural community sanitation in low- and middle-income countries: a systematic review. *International Journal of Hygiene and Environmental Health* 221: 121–33. 29133138 10.1016/j.ijheh.2017.10.018

[R26] Nussbaum M . 2001. Symposium on Amartya Sen’s philosophy: 5 adaptive preferences and women’s options. *Economics and Philosophy* 17: 67–88.

[R27] Parkin D, Devlin N. 2006. Is there a case for using visual analogue scale valuations in cost-utility analysis? *Health Economics* 15: 653–64. 16498700 10.1002/hec.1086

[R28] Patrick DL, Bush JW, Chen MM. 1973. Methods for measuring levels of well-being for a health status index. *Health Services Research* 8: 228–45. 4761617 PMC1071759

[R29] Pickard AS, Neary MP, Cella D. 2007. Estimation of minimally important differences in EQ-5D utility and VAS scores in cancer. *Health and Quality of Life Outcomes* 5: 70. 10.1186/1477-7525-5-70 PMC2248572 18154669

[R30] Pickering AJ, Djebbari H, Lopez C, Coulibaly M, Alzua ML. 2015. Effect of a community-led sanitation intervention on child diarrhoea and child growth in rural Mali: a cluster-randomised controlled trial. *The Lancet Global Health* 3: e701–1. 26475017 10.1016/S2214-109X(15)00144-8

[R31] Polit DF . 2014. Getting serious about test-retest reliability: a critique of retest research and some recommendations. *Quality of Life Research* 23: 1713–20. 24504622 10.1007/s11136-014-0632-9

[R32] Price DD, Staud R, Robinson ME. 2012. How should we use the visual analogue scale (VAS) in rehabilitation outcomes? II: Visual analogue scales as ratio scales: an alternative to the view of Kersten et al. *Journal of Rehabilitation Medicine* 44: 800–1. Discussion 803–4. 22915047 10.2340/16501977-1031 PMC3805376

[R33] Ram PK, Sahli MW, Arnold B et al. 2014. Validity of rapid measures of handwashing behavior: an analysis of data from multiple impact evaluations in the global scaling up handwashing project.

[R34] Rashidi AA, Anis AH, Marra CA. 2006. Do visual analogue scale (VAS) derived standard gamble (SG) utilities agree with Health Utilities Index utilities? A comparison of patient and community preferences for health status in rheumatoid arthritis patients. *Health and Quality of Life Outcomes* 4: 25. 10.1186/1477-7525-4-25 PMC1553436 16626489

[R35] Reeve BB, Wyrwich KW, Wu AW et al. 2013. ISOQOL recommends minimum standards for patient-reported outcome measures used in patient-centered outcomes and comparative effectiveness research. *Quality of Life Research* 22: 1889–905. 23288613 10.1007/s11136-012-0344-y

[R36] Rego R, Watson S, Gill P, Lilford R. 2022. The impact of diarrhoea measurement methods for under 5s in low- and middle-income countries on estimated diarrhoea rates at the population level: a systematic review and meta-analysis of methodological and primary empirical studies. *Tropical Medicine and International Health* 27: 347–68. 35203100 10.1111/tmi.13739 PMC9313555

[R37] Robinson A, Loomes G, Jones-Lee M. 2001. Visual analog scales, standard gambles, and relative risk aversion. *Medical Decision Making* 21: 17–27. 11206943 10.1177/0272989X0102100103

[R38] Ross I . 2022. Using water-adjusted person years to quantify the value of being water secure for an individual’s quality of life. *Water Research* 227: 119327. 10.1016/j.watres.2022.119327 36375227

[R39] Ross I, Cumming O, Dreibelbis R et al. 2021a. How does sanitation influence people’s quality of life? Qualitative research in low-income areas of Maputo, Mozambique. *Social Science & Medicine* 272: 113709. 10.1016/j.socscimed.2021.113709 PMC7938219 33517125

[R40] Ross I, Greco G, Adriano Z et al. 2022. Impact of a sanitation intervention on quality of life and mental wellbeing in low-income urban neighbourhoods of Maputo, Mozambique. *BMJ Open* 12: e062517. 10.1136/bmjopen-2022-062517 PMC9558791 36195460

[R41] Ross I, Greco G, Opondo C et al. 2021b. Measuring and valuing broader impacts in public health: development of a sanitation-related quality of life instrument in Maputo, Mozambique. *Health Economics* 31: 466–80. 34888994 10.1002/hec.4462

[R42] Russel KC, Hughes K, Roach M et al. 2019. Taking container-based sanitation to scale: opportunities and challenges. *Frontiers in Environmental Science* 7: 190.

[R43] Schelbert V, Meili D, Alam M-U et al. 2020. When is shared sanitation acceptable in low-income urban settlements? A user perspective on shared sanitation quality in Kumasi, Kisumu and Dhaka. *Journal of Water, Sanitation and Hygiene for Development* 10: 1–10.

[R44] Sclar GD, Penakalapati G, Caruso BA et al. 2018. Exploring the relationship between sanitation and mental and social well-being: a systematic review and qualitative synthesis. *Social Science & Medicine* 217: 121–34. 30316053 10.1016/j.socscimed.2018.09.016

[R45] Streiner DL, Norman GR, Cairney J. 2015. *Health Measurement Scales: A Practical Guide to Their Development and Use*, 5th edn. Oxford, UK: Oxford University Press.

[R46] Tidwell JB, Chipungu J, Chilengi R, Aunger R. 2018. Assessing peri-urban sanitation quality using a theoretically derived composite measure in Lusaka, Zambia. *Journal of Water, Sanitation and Hygiene for Development* 8: 668–78.

[R47] Tidwell JB, Nyarko KB, Ross I, Dwumfour-Asare B, Scott P. 2022. Evaluation of user experiences for the clean team Ghana container-based sanitation service in Kumasi, Ghana. *Journal of Water, Sanitation and Hygiene for Development* 12: 336–46.

[R48] Torrance GW . 1976. Social preferences for health states: an empirical evaluation of three measurement techniques. *Socio-Economic Planning Sciences* 10: 129–36.

[R49] Torrance GW, Feeny D, Furlong W. 2001. Visual analog scales: do they have a role in the measurement of preferences for health states? *Medical Decision Making* 21: 329–34. 11475389 10.1177/0272989X0102100408

[R50] Tumwebaze IK, Orach CG, Niwagaba C, Luthi C, Mosler HJ. 2013. Sanitation facilities in Kampala slums, Uganda: users’ satisfaction and determinant factors. *International Journal of Environmental Health Research* 23: 191–204. 22873693 10.1080/09603123.2012.713095

[R51] UNICEF & WHO . 2023. *Progress on Household Drinking Water, Sanitation and Hygiene 2000–2022: Special Focus on Gender*. New York, NY, USA: UNICEF & WHO.

[R52] Welsch H . 2006. Environment and happiness: valuation of air pollution using life satisfaction data. *Ecological Economics* 58: 801–13.

[R53] WHO . 1948. Constitution of The World Health Organization (978 92 4 165047 2). https://apps.who.int/gb/bd/PDF/bd47/EN/constitution-en.pdf?ua=1, accessed 25 October 2024.

[R54] Whynes DK . 2008. Correspondence between EQ-5D health state classifications and EQ VAS scores. *Health and Quality of Life Outcomes* 6: 94. 10.1186/1477-7525-6-94 PMC2588564 18992139

[R55] Wolf J, Hubbard S, Brauer M et al. 2022. Effectiveness of interventions to improve drinking water, sanitation, and handwashing with soap on risk of diarrhoeal disease in children in low-income and middle-income settings: a systematic review and meta-analysis. *The Lancet* 400: 48–59. 10.1016/S0140-6736(22)00937-0 PMC9251635 35780792

[R56] World Bank . 2019. Evaluating the Potential of Container-Based Sanitation. https://hdl.handle.net/10986/31292, accessed 25 October 2024.

