# Supplementary material for: Validity of a visual analogue scale to measure and value the perceived level of sanitation: evidence from Ghana and Mozambique
Source: Health Policy Plan. 2024 Oct 5;40(1):42–51. doi: 10.1093/heapol/czae092 (PMC11724637; doi:10.1093/heapol/czae092)
Supplement: czae092_Supp [file czae092_supp.zip › czae092_Supp/supporting information_CLEAN.docx]

# Supplementary materials

**Contents**

1. EQ-VAS
2. SanQoL-5 questions as applied in these studies
3. Additional sanitation VAS valuation tasks
4. Comparison of dropouts to non-dropouts
5. Pair-wise correlations between variables included in construct validity assessment
6. Regression output for concurrent construct validity assessments
7. Probability density distributions of VAS scores and SanQoL-5 index values
8. Regression output for responsiveness assessments
9. Version of sanitation VAS recommended in future research

# EQ-VAS

The EQ-VAS is a thermometer-like vertical line with endpoints labelled “the best health you can imagine” and “the worst health you can imagine”, denoted as 100 and 0 respectively. It is demarcated in units of one and labelled in units of 5. To be defined as the EQ-5D, a study must include the five questions comprising the EQ-5D descriptive system as well as the EQ-VAS. They should not be used independently of each other.

Figure A‑1: EuroQoL EQ-VAS


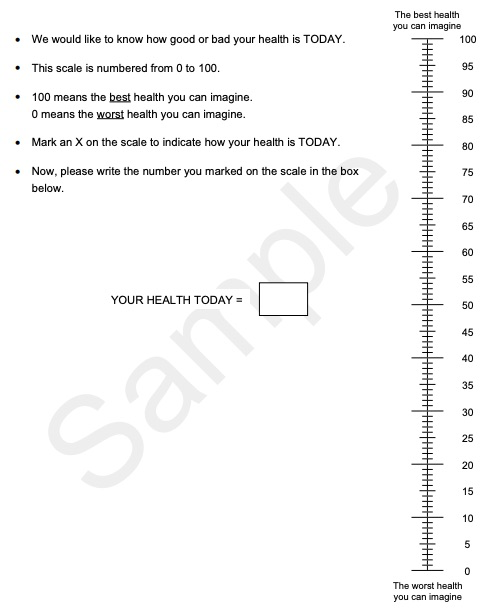


©EuroQol Research Foundation. EQ-5D™ is a trademark of the EuroQol Research Foundation” Reproduced by permission of EuroQol Research Foundation. Reproduction of this version is not allowed. For reproduction, use or modification of the EQ-5D (any version), please register your study by using the online EQ registration page: [www.euroqol.org](http://www.euroqol.org)

# SanQoL-5 questions as applied in these studies

In subsequent studies, the core SanQoL-5 questions have been updated ([www.SanQoL.org](http://www.SanQoL.org)) but those applied in Ghana and Mozambique were as per the table below. Responses are combined into a single index value ranging from 0 (worst) to 1 (best), according to the relative value of attributes elicited from the target population, such as a scoring exercise, discrete choice experiment, or similar. This valuation step underlies the claim to be measuring the value of sanitation overall, not only the status of diverse attributes.

Table B‑1: SanQoL questions (descriptive system of sanitation states)

| **Attribute** | **Questionnaire item** | **Responses** |
| --- | --- | --- |
| Disgust | Can you use the toilet without feeling disgusted? | - Always - Sometimes - Rarely - Never |
| Health | Can you use the toilet without worrying that it spreads diseases? |  |
| Privacy | Can you use the toilet in private, without being seen? |  |
| Shame | Can you use the toilet without feeling ashamed for any reason? |  |
| Safety | Are you able to feel safe while using the toilet? |  |

# Additional sanitation VAS valuation tasks

Table C‑1: VAS valuation task #2

Table C‑2: VAS valuation task #3

# Comparison of dropouts to non-dropouts

Table D‑1: Comparison of pre-intervention characteristics by dropouts and non-dropouts

|  | Dropouts | Non-dropouts (retained in analysis) | p-value in t-test |
| --- | --- | --- | --- |
| Female | 78.0% | 77.7% | 0.938 |
| Pre-intervention VAS | 5.2  (4.8-5.6) | 5.2  (4.9-5.4) | 0.915 |
| Pre-intervention SanQoL-5 index | 0.64  (0.59-0.68) | 0.59  (0.57-0.63) | 0.127 |

Table D‑2: Reasons given for dropout after installation

|  | **n** | **%** |
| --- | --- | --- |
| Space constraints | 25 | 6.2% |
| Customer relocated | 8 | 2.0% |
| Didn’t understand payment policy/complain it's expensive | 8 | 2.0% |
| Household head/partner disagreed to use | 6 | 1.5% |
| Complaints of odour issues | 3 | 0.7% |
| Only once per week toilet pick | 3 | 0.7% |
| Landlord disallowed usage | 2 | 0.5% |
| Refused to give any reason | 2 | 0.5% |
| Other reasons | 10 | 2.5% |
|  |  |  |
| sub-total of dropout after installation | 67 |  |
| total baseline respondents | 404 | 17% |

Note. This attrition (28%) was due to: customer dropout after installation (17%, shown above), customer dropout before installation (9%), and unavailability of active customers for the survey (2%)

# Pair-wise correlations between variables included in construct validity assessment

Table E‑1: Pairwise correlations in Ghana (Pearson's r)

|  | **Toilet has ceramic pan/floor** | **Handwashing facility near toilet** | **Toilet pan is not visibly dirty with faeces** | **Toilet has water seal** | **Toilet is on-compound** |
| --- | --- | --- | --- | --- | --- |
| **Toilet has ceramic pan/floor** | *1.00* |  |  |  |  |
| **Handwashing facility near toilet** | 0.45*** | *1.00* |  |  |  |
| **Toilet pan is not visibly dirty with faeces** | -0.12* | 0.08 | *1.00* |  |  |
| **Toilet has water seal** | 0.33*** | 0.52*** | 0.18*** | *1.00* |  |
| **Toilet is on-compound** | -0.16** | -0.31*** | 0.05 | -0.47*** | *1.00* |

*p<0.10 ** p<0.05 *** p<0.01

Table E‑2: Pairwise correlations in Mozambique (Pearson's r)

|  | **Toilet floor is manufactured material** | **Toilet locks from the inside** | **Enumerator does not smell faeces** | **No solid waste observed around floor** | **Toilet roof is manufactured material** |
| --- | --- | --- | --- | --- | --- |
| **Toilet floor is manufactured material** | *1.00* |  |  |  |  |
| **Toilet locks from the inside** | 0.61*** | *1.00* |  |  |  |
| **Enumerator does not smell faeces** | 0.45*** | 0.50*** | *1.00* |  |  |
| **No solid waste observed around floor** | 0.49*** | 0.38*** | 0.18*** | *1.00* |  |
| **Toilet roof is manufactured material** | 0.74*** | 0.66*** | 0.46*** | 0.51*** | *1.00* |

*p<0.10 ** p<0.05 *** p<0.01

# Regression output for construct validity assessments

Table F‑1: Output for construct validity regressions in Ghana

| **Dependent variable** | **1. VAS** | **2. VAS** | **3. VAS** | **4. VAS** | **5. VAS** | **6. VAS** | **7. VAS** | **8. VAS** | **9. VAS** |
| --- | --- | --- | --- | --- | --- | --- | --- | --- | --- |
| **Hypothesised to be associated with VAS score** | | |  |  |  |  |  |  |  |
| Toilet has ceramic pan/floor | 0.23 | 0.87* |  |  |  |  |  |  |  |
|  | (0.50) | (0.44) |  |  |  |  |  |  |  |
| Handwashing facility near toilet | 1.25*** |  | 1.64*** |  |  |  |  |  |  |
|  | (0.31) |  | (0.19) |  |  |  |  |  |  |
| Toilet pan is not visibly dirty with faeces | 0.82* |  |  | 1.12** |  |  |  |  |  |
|  | (0.43) |  |  | (0.42) |  |  |  |  |  |
| Toilet has water seal | 0.78** |  |  |  | 1.49*** |  |  |  |  |
|  | (0.34) |  |  |  | (0.29) |  |  |  |  |
| Toilet is on-compound | 0.71 |  |  |  |  | -0.47 |  |  |  |
|  | (0.40) |  |  |  |  | (0.39) |  |  |  |
| **Negative controls** |  |  |  |  |  |  |  |  |  |
| Years in dwelling |  |  |  |  |  |  | -0.19 |  |  |
|  |  |  |  |  |  |  | (0.13) |  |  |
| Education |  |  |  |  |  |  |  | 0.23 |  |
|  |  |  |  |  |  |  |  | (0.22) |  |
| Partner |  |  |  |  |  |  |  |  | -0.59 |
|  |  |  |  |  |  |  |  |  | (0.38) |
| Constant | 3.21*** | 4.96*** | 4.37*** | 4.43*** | 4.21*** | 5.37*** | 6.08*** | 5.08*** | 5.60*** |
|  | (0.43) | (0.25) | (0.16) | (0.42) | (0.33) | (0.15) | (0.66) | (0.18) | (0.35) |
| Observations | 213 | 215 | 215 | 215 | 215 | 270 | 291 | 280 | 280 |

Standard errors in parentheses. *p<0.10 ** p<0.05 *** p<0.01

Table F‑2: Table 6: Output for construct validity regressions in Mozambique

| **Dependent variable** | **1. VAS** | **2. VAS** | **3. VAS** | **4. VAS** | **5. VAS** | **6. VAS** | **7. VAS** | **8. VAS** | **9. VAS** |
| --- | --- | --- | --- | --- | --- | --- | --- | --- | --- |
| **Hypothesised to be associated with VAS score** | | |  |  |  |  |  |  |  |
| Toilet floor material is solid | 0.99*** | 2.74*** |  |  |  |  |  |  |  |
|  | (0.37) | (0.25) |  |  |  |  |  |  |  |
| Toilet has inside lock | 0.87*** |  | 2.51*** |  |  |  |  |  |  |
|  | (0.32) |  | (0.25) |  |  |  |  |  |  |
| No strong smell of faeces | 0.73** |  |  | 2.04*** |  |  |  |  |  |
|  | (0.28) |  |  | (0.25) |  |  |  |  |  |
| No solid waste observed | 0.83*** |  |  |  | 2.30*** |  |  |  |  |
|  | (0.32) |  |  |  | (0.32) |  |  |  |  |
| Toilet has solid roof | 0.66* |  |  |  |  | 2.66*** |  |  |  |
|  | (0.36) |  |  |  |  | (0.26) |  |  |  |
| **Negative controls** |  |  |  |  |  |  |  |  |  |
| Years in dwelling |  |  |  |  |  |  | 0.15 |  |  |
|  |  |  |  |  |  |  | (0.21) |  |  |
| Education |  |  |  |  |  |  |  | 0.28 |  |
|  |  |  |  |  |  |  |  | (0.26) |  |
| Partner |  |  |  |  |  |  |  |  | -0.04 |
|  |  |  |  |  |  |  |  |  | (0.24) |
| Constant | 3.14*** | 3.85*** | 4.52*** | 4.39*** | 3.87*** | 4.05*** | 4.77*** | 5.46*** | 5.65*** |
|  | (0.28) | (0.19) | (0.17) | (0.17) | (0.28) | (0.20) | (1.24) | (0.22) | (0.19) |
| Observations | 420 | 421 | 424 | 421 | 421 | 420 | 424 | 424 | 424 |

# Distributions of VAS scores and SanQoL index values

Figure G‑1: Probability density distributions of VAS scores and SanQoL index values by treatment group

|  | **Ghana** | **Mozambique** |
| --- | --- | --- |
| **VAS scores on x-axis** | 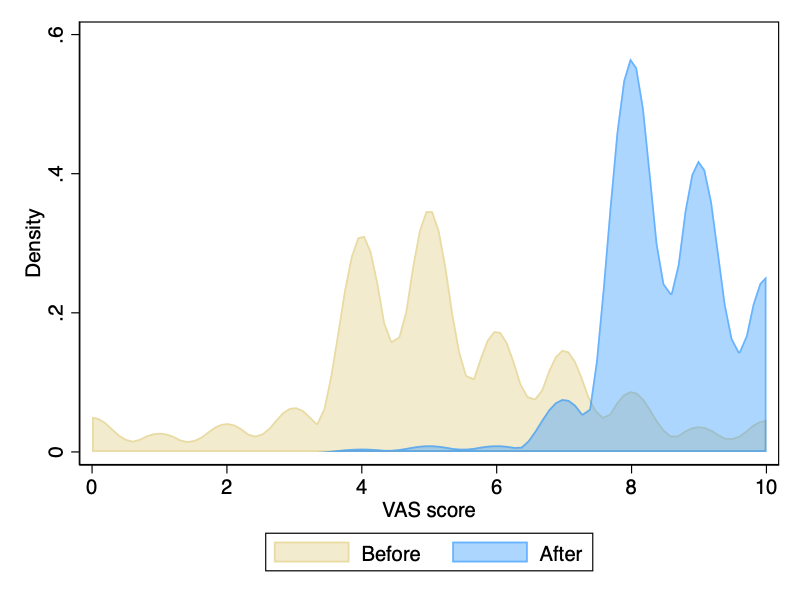 | 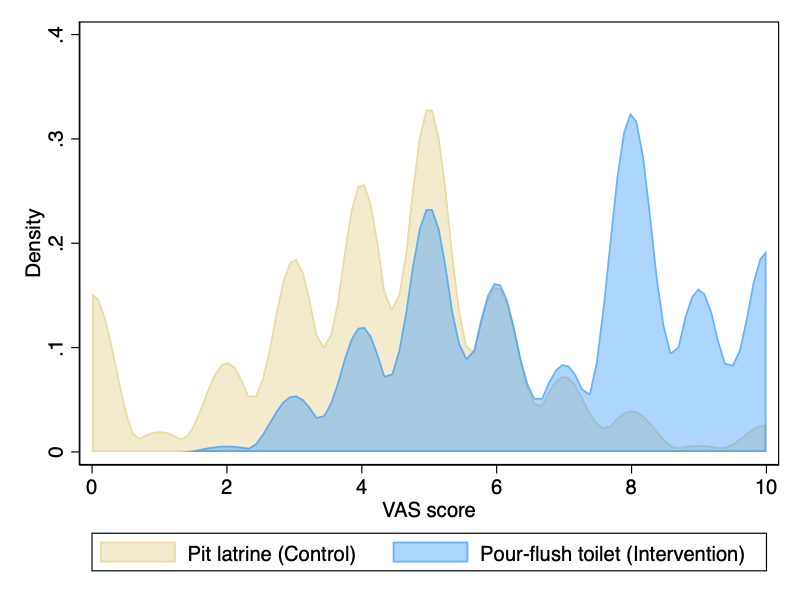 |
| **SanQoL index values on x-axis** | 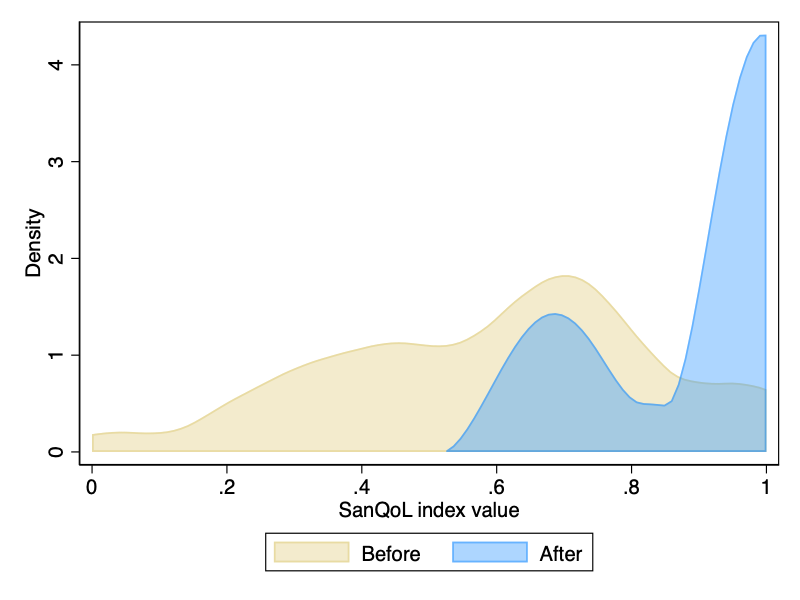 | 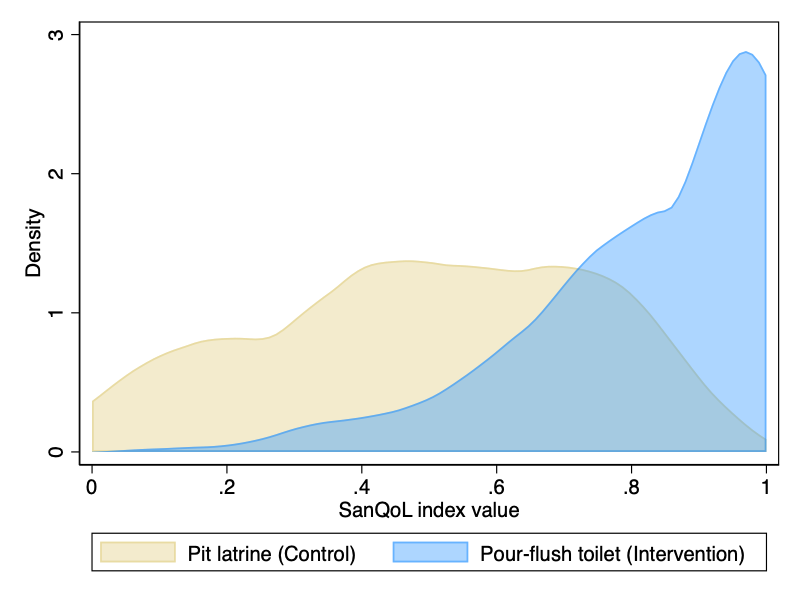 |

Figure G‑2: Frequency of VAS scores in samples overall (not by treatment group)

|  | **Ghana** | **Mozambique** |
| --- | --- | --- |
|  | 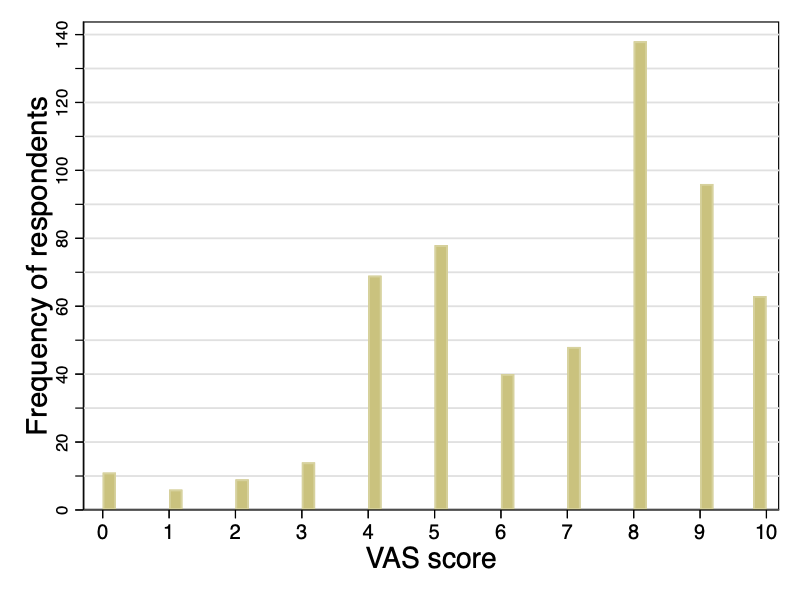 | 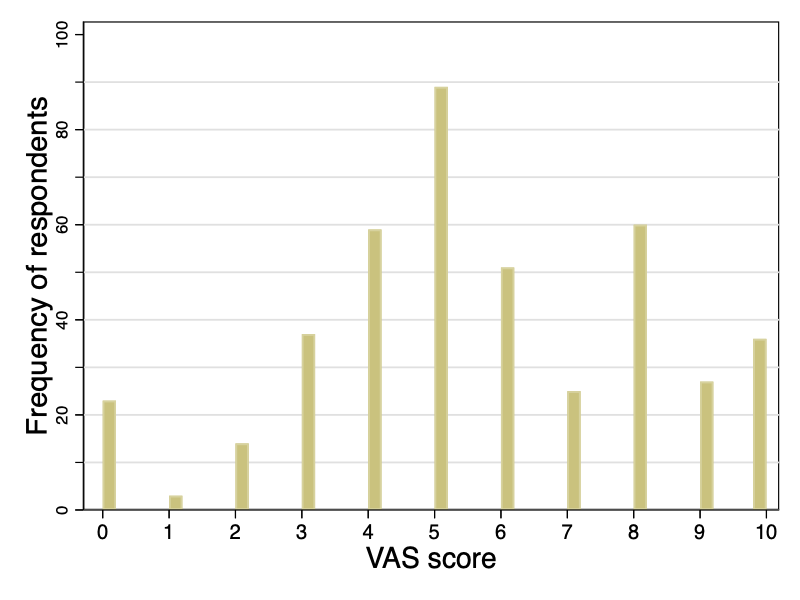 |

# Regression output for responsiveness assessments

Table H‑1: Output for GLMM assessing responsiveness in (baseline and endline data in Ghana)

|  | **Ghana** | **Mozambique** |
| --- | --- | --- |
| Intervention | 3.42*** | 2.91*** |
|  | (0.11) | (0.24) |
| Aged 60+ | 0.49* | -0.15 |
|  | (0.26) | (0.27) |
| Female | 0.04 | -0.30* |
|  | (0.11) | (0.16) |
| Wealth index | 0.24*** | -0.07 |
|  | (0.08) | (0.10) |
| Constant | 5.07*** | 4.28*** |
|  | (0.13) | (0.20) |
| Observations | 560 | 423 |

Dependent variable is VAS score. Standard errors in parentheses

*p<0.10 ** p<0.05 *** p<0.01

# Version of sanitation VAS recommended in future research

Figure I‑1: version of VAS recommended for use in future research

Nb. if a participant struggles to understand the above, they can instead be shown the simplified version overleaf, and fieldworker translates the response to the 0-100 scale.

Figure I‑2: version of VAS for respondents who struggle with the 0-100 version

Fieldworker: if the participant struggles to understand the 0-100 version above, they can instead be shown the simplified version here. Ask the participant to consider the cartoon faces, and choose which corresponds to how they feel about their level of sanitation today.
