# Supplementary material for: Validity of a visual analogue scale to measure and value the perceived level of sanitation: evidence from Ghana and Mozambique
Source: Health Policy Plan. 2024 Oct 5;40(1):42–51. doi: 10.1093/heapol/czae092 (PMC11724637; doi:10.1093/heapol/czae092)
Supplement: czae092_Supp [file czae092_supp.zip › czae092_Supp/Table 1_update.docx]

|  | **Kumasi** | **Mozambique** | **Direction of hypothesised association**  **(and rationale)** |
| --- | --- | --- | --- |
| **Hypothesised to be associated with VAS score** | | | |
| **Floor/slab** | Toilet has ceramic pan/floor | Toilet floor is manufactured material* | Positive (more modern and easier to keep clean compared to lower-quality floors) |
| **Water seal** | Toilet has water seal | *n/a (100% collinearity with intervention)* | Positive (keep out smells and flies compared to direct-drop pit latrines) |
| **Roof** | *n/a (96% have)*** | Toilet roof is manufactured material* | Positive (stops people looking in from above and prevents rain entering) |
| **Lock** | *n/a (97% have) *** | Toilet locks from the inside | Positive (stops others entering, by mistake or on purpose) |
| **Cleanliness** | Toilet pan is not visibly dirty with faeces | Enumerator does not smell faeces | Positive (less disgusting to use) |
| **Solid waste** | *n/a (not collected)* | No solid waste observed around floor | Positive (less disgusting to use). nb. solid waste referred to waste other than anal cleansing materials |
| **On-compound** | Toilet is on-compound | *n/a (100% on-compound)* | Positive (easier and quicker to access, with more safety and less worry) |
| **Handwashing** | Handwashing facility near toilet | *n/a (not collected)* | Positive (easier to feel clean after using the toilet) |
| **Negative controls (hypothesised not to be associated with VAS score)** | | | |
| **Years in dwelling** | Respondent years lived in that dwelling | | n/a (no obvious rationale for association with VAS score) |
| **Education** | Respondent completed primary education | |  |
| **Partner** | Respondent has a partner | |  |
