# Supplementary material for: Validity of a visual analogue scale to measure and value the perceived level of sanitation: evidence from Ghana and Mozambique
Source: Health Policy Plan. 2024 Oct 5;40(1):42–51. doi: 10.1093/heapol/czae092 (PMC11724637; doi:10.1093/heapol/czae092)
Supplement: czae092_Supp [file czae092_supp.zip › czae092_Supp/Table 2_update.docx]

|  | **Ghana at baseline (n=291)** | **Mozambique**  **(n=424)** |
| --- | --- | --- |
| **Respondent demographic characteristics** | | |
| Respondent is female | 215 (77%) | 220 (52%) |
| Respondent mean age | 44.1 (12.7) | 39.9 (15.3) |
| Aged 18-29 | 14 (5%) | 126 (30%) |
| Aged 30-44 | 147 (51%) | 155 (37%) |
| Aged 45-59 | 84 (29%) | 88 (21%) |
| Aged 60+ | 46 (16%) | 55 (13%) |
| Household size | 3.2 (1.7) | 5.1 (3.0) |
| Completed primary school or above | 192 (67%) | 268 (63%) |
| Piped water on-premises | 63 (23%) | 416 (98%) |
| **Sanitation characteristics** | | |
| *Type of toilet* | | |
| Flush or pour-flush toilet | 223 (83%) | 222 (52%) |
| Pit latrine | 47 (17%) | 202 (48%) |
| *Nature of sharing* | | |
| Not shared with other households | 13 (5%) | 47 (11%) |
| Shared but not public toilet | 62 (23%) | 377 (89%) |
| Public toilet | 195 (72%) | 0 (0%) |
| Toilet is on-plot | 42 (16%) | 416 (98%) |
| Toilet has solid walls | 205 (74%) | 275 (65%) |
| Toilet has inside lock | 182 (66%) | 187 (44%) |
