# Supplementary material for: Validity of a visual analogue scale to measure and value the perceived level of sanitation: evidence from Ghana and Mozambique
Source: Health Policy Plan. 2024 Oct 5;40(1):42–51. doi: 10.1093/heapol/czae092 (PMC11724637; doi:10.1093/heapol/czae092)
Supplement: czae092_Supp [file czae092_supp.zip › czae092_Supp/Table 3_update.docx]

|  | **Ghana** | | **Mozambique** | |
| --- | --- | --- | --- | --- |
|  | **individual** | **concurrent** | **individual** | **concurrent** |
| **Hypothesised to be associated with VAS score** | | | | |
| **Floor/slab** | 0.054* | 0.852 | <0.001*** | 0.007*** |
| **Water seal** | 0.006*** | 0.040** | *n/a* | *n/a* |
| **Roof** | *n/a* | *n/a* | <0.001*** | 0.069* |
| **Lock** | *n/a* | *n/a* | <0.001*** | 0.007*** |
| **Cleanliness** | 0.004*** | 0.112 | <0.001*** | 0.010** |
| **Solid waste** | *n/a* | *n/a* | <0.001*** | 0.009*** |
| **On-compound** | 0.214 | 0.066* | *n/a* | *n/a* |
| **Handwashing** | <0.001*** | <0.001*** | *n/a* | *n/a* |
| **R^2^** |  | 22% |  | 34% |
| **Negative controls** | | | | |
| **Years in dwelling** | 0.266 | *n/a* | 0.484 | *n/a* |
| **Education** | 0.312 | *n/a* | 0.295 | *n/a* |
| **Partner** | 0.232 | *n/a* | 0.854 | *n/a* |
