# Supplementary material for: Validity of a visual analogue scale to measure and value the perceived level of sanitation: evidence from Ghana and Mozambique
Source: Health Policy Plan. 2024 Oct 5;40(1):42–51. doi: 10.1093/heapol/czae092 (PMC11724637; doi:10.1093/heapol/czae092)
Supplement: czae092_Supp [file czae092_supp.zip › czae092_Supp/Table 4_update.docx]

|  |  | **Ghana (n=280)** | | **Mozambique (n=424)** | |
| --- | --- | --- | --- | --- | --- |
|  |  | **without intervention** | **with intervention** | **without intervention** | **with intervention** |
| **Sample size** | | 280 (before) | 280 (after) | 202 (control) | 222 (int'n) |
| **Mean VAS score** | Mean | 5.1 | 8.6 | 4.1 | 7.0 |
|  | (s.e.) | (0.1) | (0.1) | (0.2) | (0.1) |
| **Unadjusted**  **models** | Unadjusted diff. (95% CI) | 3.4*** (3.2 - 3.6) | | 2.9*** (2.4 - 3.4) | |
|  | p-value | <0.001 | | <0.001 | |
| **Adjusted models** | Adjusted diff. (95% CI) | 3.4*** (3.2 - 3.6) | | 2.9*** (2.4 - 3.4) | |
|  | p-value | <0.001 | | <0.001 | |
|  | Effect size (Cohen’s d) | 2.1 SD | | 1.3 SD | |
