# Supplementary material for: Validity of a visual analogue scale to measure and value the perceived level of sanitation: evidence from Ghana and Mozambique
Source: Health Policy Plan. 2024 Oct 5;40(1):42–51. doi: 10.1093/heapol/czae092 (PMC11724637; doi:10.1093/heapol/czae092)
Supplement: czae092_Supp [file czae092_supp.zip › czae092_Supp/Table 5_update.docx]

|  | **State 1** | **State 2** | **State 3** |
| --- | --- | --- | --- |
| Disgust | rarely | Never | sometimes |
| Disease | never | Never | sometimes |
| Privacy | rarely | sometimes | always |
| Shame | never | Never | sometimes |
| Safety | rarely | always | rarely |
| mean | 4.2 | 4.4 | 5.5 |
| (95% CI) | (4.1-4.3) | (4.2 - 4.5) | (5.1 - 5.8) |
