# Supplementary figures and images for: Validity of a visual analogue scale to measure and value the perceived level of sanitation: evidence from Ghana and Mozambique
Source: Health Policy Plan. 2024 Oct 5;40(1):42–51. doi: 10.1093/heapol/czae092 (PMC11724637; doi:10.1093/heapol/czae092)

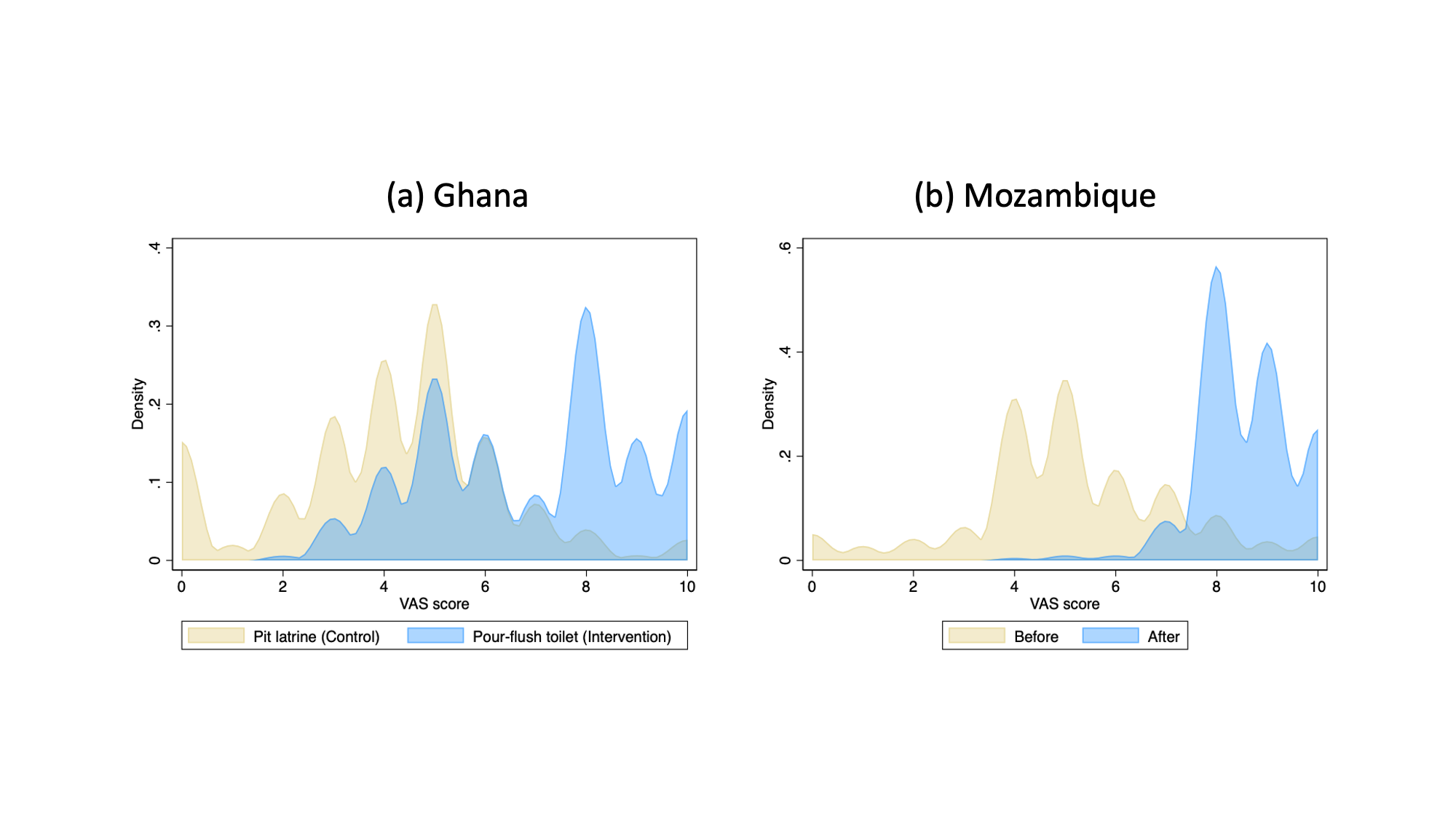

Supplement: czae092_Supp [file czae092_supp.zip › czae092_Supp/fig3 - edit.tiff]

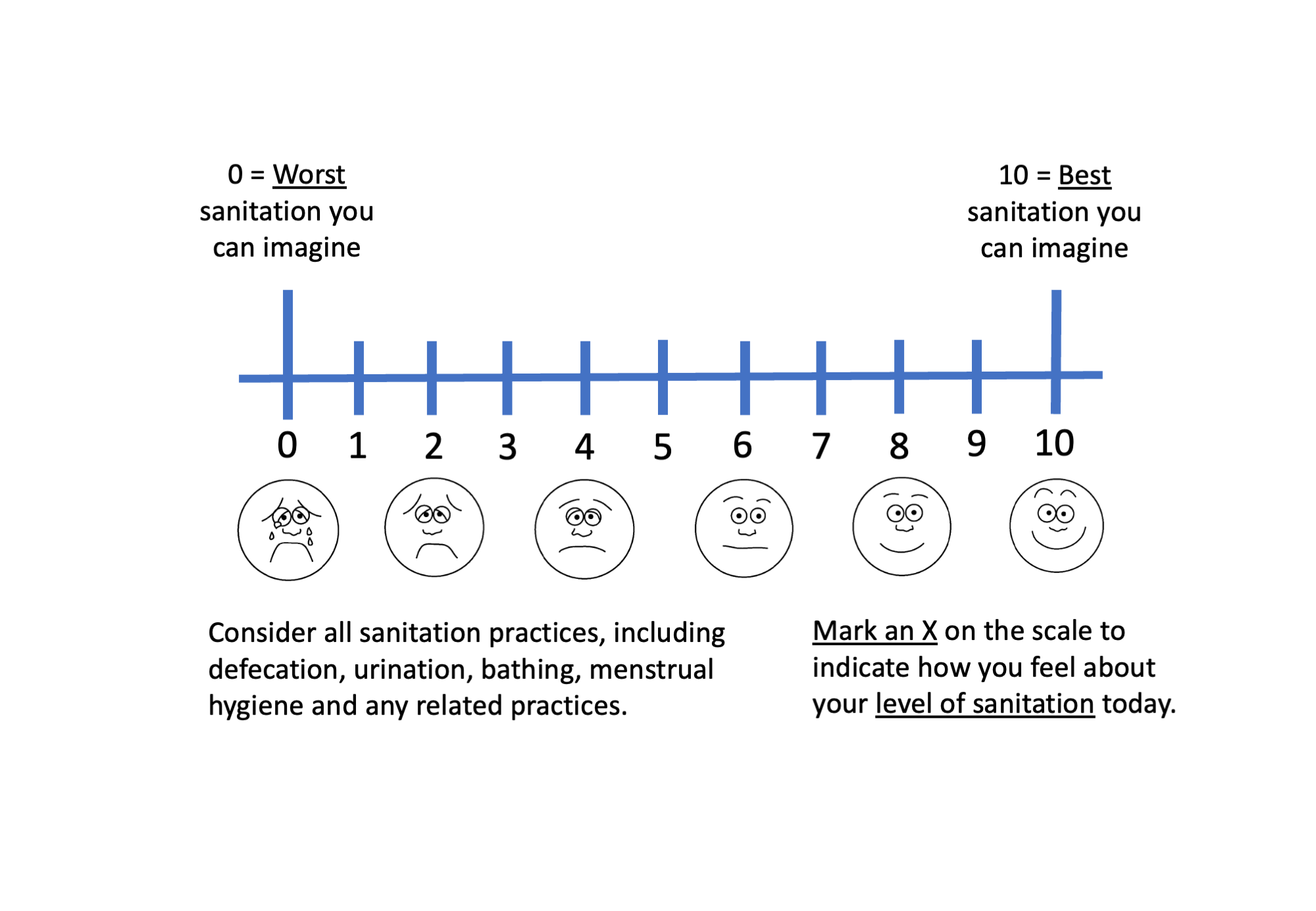

Supplement: czae092_Supp [file czae092_supp.zip › czae092_Supp/VAS Fig1 - VAS.tiff]

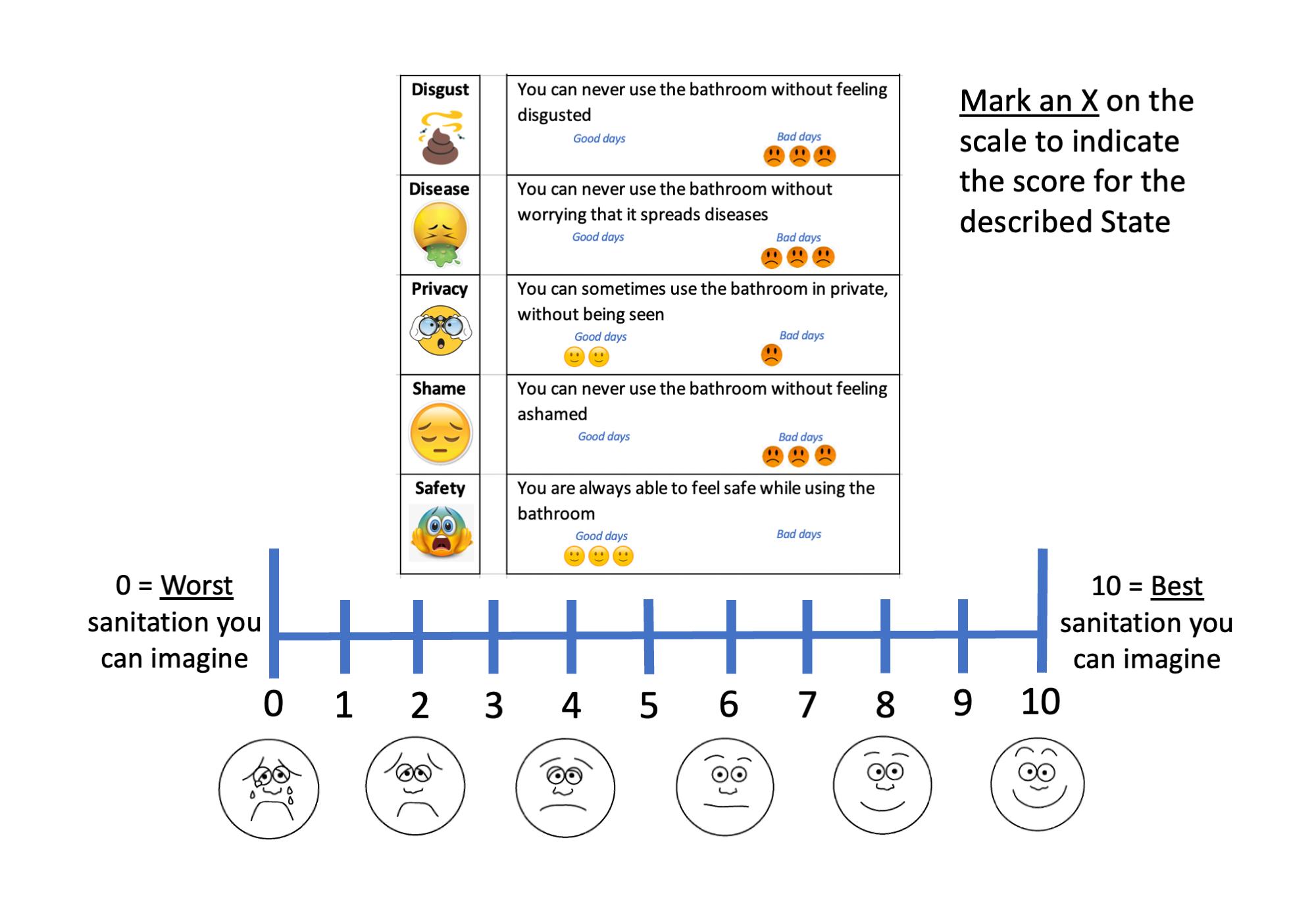

Supplement: czae092_Supp [file czae092_supp.zip › czae092_Supp/VAS Fig2 - valuation task.tiff]
